# Exploitation of thermal gradients for investigation of irradiation temperature effects with charged particles

**DOI:** 10.1038/s41598-019-49585-0

**Published:** 2019-09-19

**Authors:** Chris D. Hardie, Andrew J. London, Joven J. H. Lim, Rob Bamber, Tonči Tadić, Marin Vukšić, Stjepko Fazinić

**Affiliations:** 1 United Kingdom Atomic Energy Authority, Culham Centre for Fusion Energy, Culham Science Centre, Abingdon, Oxon OX14 3DB UK; 2 Ruđer Bošković Institute, Bijenička c.54, 10000 Zagreb, Croatia

**Keywords:** Mechanical engineering, Mechanical properties, Techniques and instrumentation

## Abstract

The effects of radiation damage on materials are strongly dependant on temperature, making it arguably the most significant parameter of concern in nuclear engineering. Owing to the challenges and expense of irradiating and testing materials, material property data is often limited to few irradiation conditions and material variants. A new technique has been developed which enables the investigation of radiation damage of samples subject to a thermal gradient, whereby a wealth of data over a range of irradiation temperatures is produced from a single irradiation experiment. The results produced are practically inaccessible by use of multiple conventional isothermal irradiations. We present a precipitation-hardened copper alloy (CuCrZr) case-study irradiated with a linear temperature gradient between 125 and 440 °C. Subsequent micro-scale post irradiation characterisation (nanoindentation, transmission electron microscopy and atom probe tomography) highlight the capability to observe mechanical and microstructural changes over a wide range of irradiation temperatures. We observed irradiation-softening in CuCrZr that did not occur due to irradiation-enhanced aging of the Cr-precipitates. Excellent reproducibility of the new technique was demonstrated and replicated irradiation-hardening data from several isothermal neutron irradiation studies. Our new technique provides this data at a fraction of the time and cost required by conventional irradiation experiments.

## Introduction

For a vast range of applications, temperature is a primary factor concerning engineers when considering material performance in service and nuclear applications are exemplar to this. Materials subject to elevated temperatures often exhibit changes in physical and mechanical properties resulting from diffusion mechanisms resulting in degradation such as creep, ageing, phase transformations and corrosion^1^. The operational temperature range of materials is important for the economics of fission and fusion power^2–4^ and is limited for each material, generally due to irradiation hardening and embrittlement at low temperatures and creep at high temperatures^5^.

As with many industrial applications, the performance of materials in a nuclear environment is further complicated by synergistic effects from multiple conditions including irradiation. The parameter space required by theoreticians and engineers is often vast for experimental treatment, resulting in a lack of data or prohibitively expensive research campaigns^6^. Irradiation using ions instead of neutrons is one way of increasing the throughput of materials testing^7^, even so, usually only a few temperature measurements are made^8^. For qualifying materials and developing new materials, the irradiation resistance as a function of temperature is of key importance^9,10^.

This paper describes a new approach that utilises subjecting materials with a thermal gradient along the sample length^11,12^ to charged particle irradiation within an ion beam accelerator. In comparison with conventional isothermal irradiations^13,14^, this technique provides samples for post irradiation experimentation (PIE) that include a large irradiation temperature range of interest from a single irradiation experiment with a fine thermal resolution. The technique is demonstrated with micro-scale PIE techniques including nanoindentation, transmission electron microscopy (TEM) and atom probe tomography (APT) to identify trends in irradiation hardening and targeted microstructural characterisation. The current study focuses on a CuCrZr alloy, which is a high heat flux structural candidate material for nuclear fusion applications, in order to demonstrate the technique on a relevant material with particularly high thermal conductivity, where achieving the required thermal gradient is most challenging. Additionally, this material exhibits a transition from irradiation hardening to irradiation softening at ~290 °C when subject to neutron irradiation^15^, thus the irradiation response should vary substantially in the temperature range of interest.

## Results

### Technique demonstration

**Figure 1:**
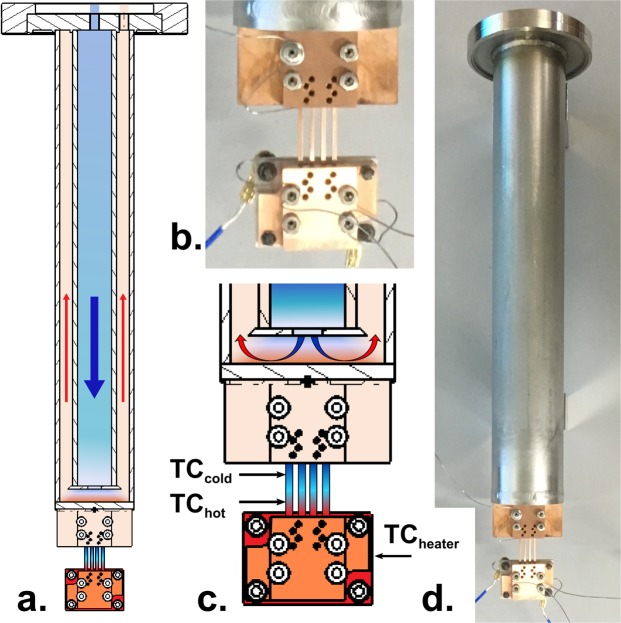
Images showing experimental setup including (**a**) a schematic diagram of the entire assembly, (**b**) photograph of the clamped samples, (**c**) larger scale schematic of the sample clamping assembly and location of thermocouples (labelled TC) and (**d**) a photograph of the entire experimental assembly. Arrows and colours indicative of water coolant flow direction and relative temperature.

Four identical CuCrZr samples were exposed to a dose of 0.2 dpa using a constant applied thermal gradient using the equipment shown in Fig. 1. A linear gradient of 125 °C to 440 °C (19 °C/mm) across the exposed area was achieved, as measured by multiple thermocouples and thermal imaging camera. More details are provided in the Methods: Irradiation section.

### Nanohardness

**Figure 2:**
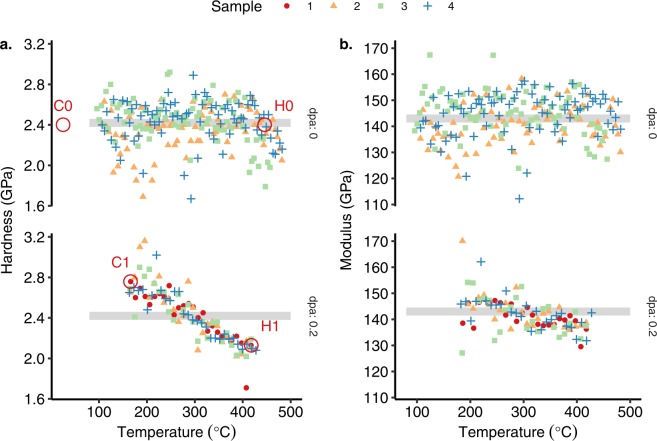
(**a**) Nanohardness and (**b**) modulus, of four samples irradiated simultaneously. Top panel shows the non-irradiated and bottom panel shows the results from the irradiated surface. The red labels in (**a**) show which indents lift-outs were taken from for the four sub-samples C0, C1, H1 and H0. The horizontal grey band shows the value of the as-received material for comparison.

The average hardness and modulus from a depth of 100 to 200 nm measured on the irradiated surface for all four samples is shown in Fig. 2. In addition to indenting the irradiated surface, 3 of the samples (no. 2–4) were polished and indented on the non-irradiated side of the sample to show the effect of the heat treatment experienced during the irradiation experiment (top graphs of Fig. 2). The hardness of the un-irradiated side of samples 2–4 (at the location T < 350 °C) was constant at 2.42 ± 0.22 GPa (one standard deviation, N = 154). At a temperature greater than 400 °C, the average hardness of the un-irradiated material was found to decrease as a function of temperature. The elastic modulus of the un-irradiated material is consistent for all positions and temperature history with an average of 143 GPa. This compares well with the value of Young’s modulus of 127.5 GPa which is given in the ITER material properties handbook for a similar grade alloy^16^.

After irradiation significant irradiation hardening was observed at low irradiation temperatures of T_irr_ < 200 °C, which decreases as a function of irradiation temperature transitioning to softening for T_irr_ > 294 °C. As shown on Table 1, all 4 samples show a near identical response with an average transition temperature, T_x_, from hardening to softening of 294 ± 23 °C (95% CI) and a gradient of –3 MPa/°C. This demonstrates that there is good reproducibility of the thermal gradient across all samples within the clamp. There is more variation in hardness in the un-irradiated material compared to the irradiated material; this reflects the microstructure which is heterogeneous on the scale of the nanoindentation volume due to relatively large grains (mean diameter of 74.5 µm as reported elsewhere^17^) and sporadic micro-metre sized CrZr particles. Following irradiation, the microstructure is dominated by the irradiation damage which is homogeneous at a smaller scale, leading to reduced variation in hardness. Ringed data in Fig. 2a shows where, and therefore what temperature, samples were taken for further microstructural investigation. The elastic modulus of the irradiated material was similar to the un-irradiated material. A slight decrease in modulus with irradiation temperature may be apparent from the data, however this was likely due to the enhanced pile-up surrounding the indenter tip following irradiation at cold temperatures, which decreases with increasing irradiation temperature, as observed elsewhere^18^.

**Table 1:** Linear fit data for hardness versus irradiation temperature for each sample and the average for all samples. Uncertainty bounds are given by a 95% CI.

Sample	Intercept (GPa)	Gradient (GPa/°C)	T_x_ (°C)
1	0.80 ± 0.08	−0.0028 ± 0.0003	290 ± 43
2	1.05 ± 0.13	−0.0035 ± 0.0004	301 ± 51
3	0.87 ± 0.10	−0.0030 ± 0.0003	291 ± 47
4	0.81 ± 0.08	−0.0028 ± 0.0003	294 ± 39
*All*	*0.88* ± *0.05*	*−0.0030* ± *0.0002*	294 ± 23

The intercept value is taken at T_irr_ = 0 °C.

**Figure 3:**
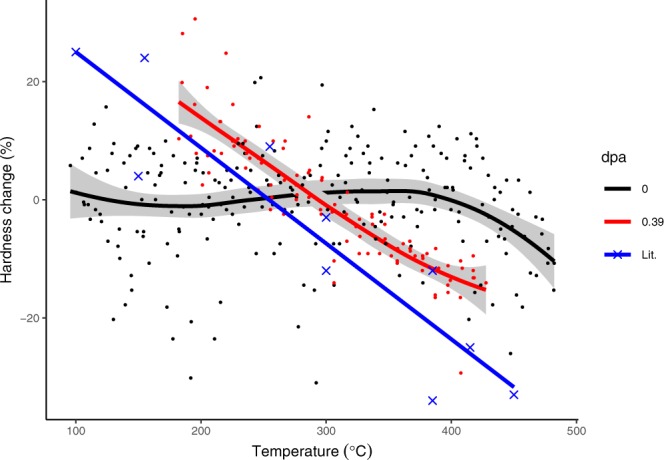
Change in nanohardness as function of irradiation temperature. Black line shows the side of the sample annealed during the irradiation but not irradiated. Red line shows the irradiated surface. Fit is given by local regression, grey band indicates 95% confidence interval for fit. Blue line and crosses are literature data^15^.

The data from Fig. 2 is fitted using a local regression (75% smoothing span width) in Fig. 3. The grey band shows a 95% confidence interval for the fitted line. The non-irradiated tests do not show any softening until above 400 °C, whereas the softening of the irradiated specimens is clear above 300 °C. Figure 3 also shows a comparison with the change in uniaxial tensile yield strength for neutron irradiated Cu-alloys. These literature data are taken from ref.^15^. All the literature results presented in Fig. 3 are from a range of different alloys (0.5–0.9% Cr, 0.1–0.2% Zr, 0–0.1% Mg), different reactors and different total doses (3–30 dpa, ~10^−7^ dpa/s). The test temperature in each case was equal to the irradiation temperature which will give lower values at higher temperatures compared to testing at room temperature.

### Microstructural investigation: TEM

**Figure 4:**
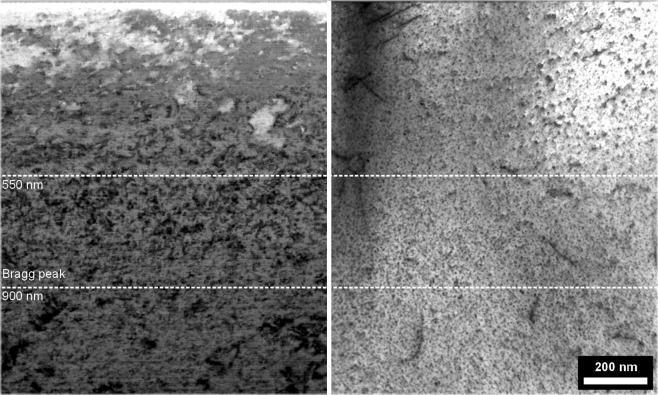
C1 sample (left) and H1 sample (right), bright field TEM image showing the full depth of the ion-implanted layer. The Bragg peak has a SRIM damage-dose of 0.2 dpa.

Microstructural investigations can be made on the micrometre scale by using the FIB lift-out technique. Electron transparent cross-section samples were made from 4 specimen regions, C0, C1, H1 and H0. Low-magnification images are shown in Fig. 4, for both the C1 sample (0.2 dpa, 166 °C) and H1 (0.2 dpa, 418 °C) sample. At the depth of 550–900 nm, a dense region of defects can be seen at the depth predicted as the peak-damage region by SRIM. The damage defects were not quantified in detail, however EDS was used to observe the particle distribution in each of the 4 samples.

**Figure 5:**
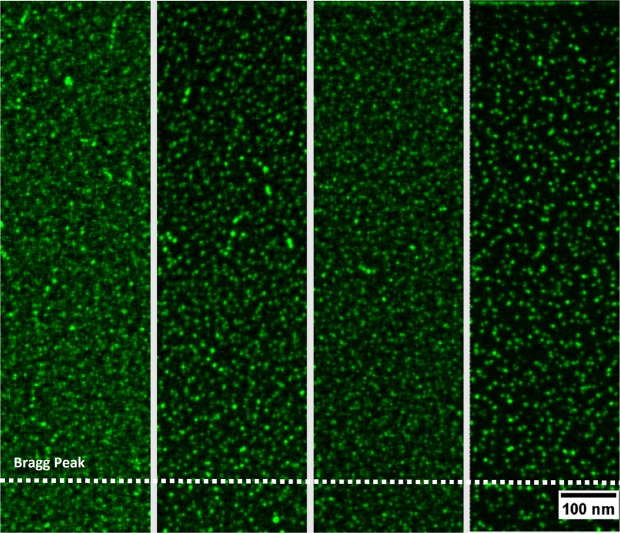
Integrated Cr EDS signal for C0, C1, H1 and H0; treatment temperatures 25, 166, 418 and 446 °C. The top of the image is the surface of the sample and the Bragg peak depth (~900 nm) is labelled near the bottom of the image. Only C1 and H1 were irradiated (0.2 dpa). Contrast adjusted.

Figure 5 shows the Cr-Kα EDS spectrum images from C0, C1, H1 and H0. C0 is equivalent to the as-received condition as it did not exceed 100 °C and was not irradiated. Figure 5 shows that qualitatively the particle size of the C0, C1 and H1 samples are similar, and H0 has a slightly larger particle size. A 1 nm step size was used to give maps showing the overall homogeneity of the particle distribution in each sample. The small particle size complicated accurate measurement of the size by STEM EDS. The EDS maps in Fig. 5 indicate that the H0 sample also has a slightly lower density of particles compared to the other 3 conditions. However, the foil thickness was not quantified therefore no conclusion can be drawn from the observed area density. The particles show no noticeable difference as a function of depth and therefore irradiation dose. The particle size and composition was characterised in more detail using atom probe tomography.

### Microstructural investigation: APT

**Figure 6:**
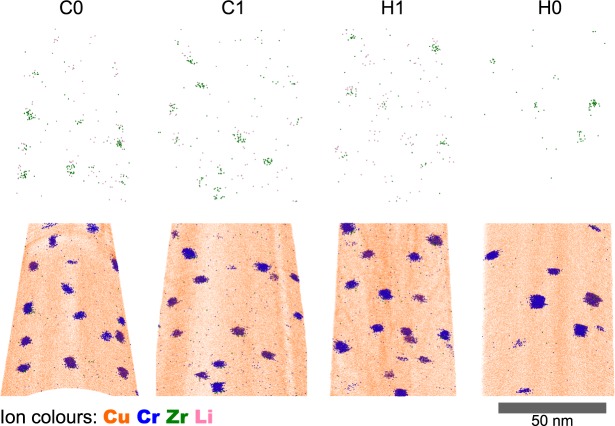
10 nm thick slices of reconstructed atom probe data. One sample volume from each of the irradiation conditions, C/H for cold/hot and 1/0 for 0.2 and 0 dpa. Zr and Li ions are shown separately for clarity.

At least two of every condition cold un-irradiated C0, cold irradiated C1, hot irradiated H1 and hot un-irradiated H0 were successfully analysed by atom probe. A representative sample of each condition is shown in Fig. 6. As noted above, the C0 specimen is equivalent to the as heat-treated material, which contains a high number density of Cr-rich particles in a low-Cr Cu matrix. The other irradiated and heated analysed volumes similarly show a high number density of Cr-rich particles. The small size of the particles results in a high content of Cu detected in the particles (76 ± 1 at%) due to trajectory aberrations^19^. The average particle compositions for all the samples are: Cr 24 ± 1, Zr 0.15 ± 0.05, Li 0.05 ± 0.01 at%, where the uncertainty is given by the standard error of the mean (N = 10). Therefore the Cr/Zr ratio in the particles is ~150. However, even within one experimental volume, the particle composition had a large variance and some had dependence on particle size. There were negligible changes in the particle compositions as a function of irradiation condition, except C1 which had lower Zr and Fe levels. The compositions of the volume once the particles were removed were similar in all conditions: Cr 0.027 ± 0.004 at%, Zr 0.018 ± 0.004 at% and Li 0.002 ± 0.001 at% (±standard error of the mean, N = 10). The low content of Cr in the matrix agrees with negligible solubility of Cr at the original aging temperature^20^.

**Figure 7:**
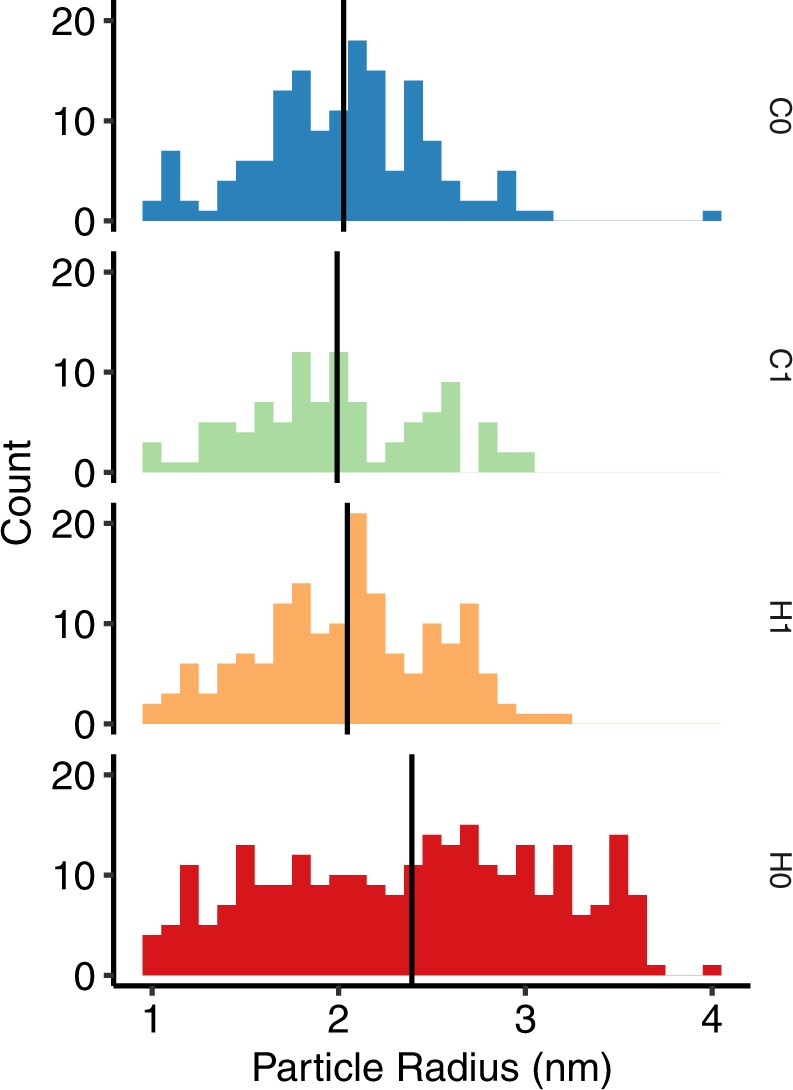
Particle radius histograms for each sample condition. Measured particle radius is calculated from the number of Cr atoms (see Methods section). Black vertical line marks the mean particle radius.

The particle size distributions are shown in Fig. 7. There is no significant change in particle radius until the temperature exceeds 440 °C as found in the H0 lift-out sample. H0 also has a lower particle number density 1.5–1.8 × 10^23^ m^−3^ compared to 2.1–2.9 × 10^23^ m^−3^ for C0, C1 and H1; range shown is a 90% confidence interval based on counting statistics.

## Discussion

The production of a large thermal gradient on small samples as described here was challenging. Conceptual design and analysis suggested that the primary factors influencing the thermal gradient include both the thermal transfer across the interface between the sample and clamp at both the hot and cold ends and the sample resistance itself. The latter influences the thermal gradient by conduction using Fourier’s law of conduction in one dimension, with $Q$, $L$, $k$ and $A$ representing heat flow (per sample), length, thermal conductivity and area respectively:

$$\Delta T=\frac{QL}{kA}$$

The thermal conductivity of CuCrZr alloy is relatively temperature independent varying from 333 at 100 °C to 346 W/mK at 450 °C^16^, therefore at the temperatures studied here the gradient is practically linear. The thermal conductivity is relatively high, providing a particularly challenging material to demonstrate the capability of the technique. Practically all structural and functional materials relevant to nuclear and accelerator communities have a lower thermal conductivity and could be studied with large thermal gradients using this technique. Care is required when irradiating materials at temperatures higher than those studied here, where radiative losses become significant. Data from a third thermocouple should be used to fit the temperature profile along the sample where non-linear temperature gradients are expected.

Thermal conductivity of many materials has been shown to change as a result of irradiation due to lattice damage and chemical composition changes due to transmutation in the case of neutron irradiation. The thermal conductivity of CuCrZr has been shown to decrease following irradiation by ~10% due to displacement damage alone^21^. Here, changes in thermal conductivity as a result of ion-irradiation of a 1 µm surface layer are not expected to alter the thermal gradient because most of the thermal transport was being supported by the 1 mm thick un-irradiated substrate.

A sample cross-sectional area of approximately 1 × 1.2 mm^2^ and a sample length of 35 mm was chosen as a compromise between increasing the thermal resistance along the sample whilst reducing of risk of damage during handling. The heater assembly weighs <55 g and is supported by the samples during irradiation; this subjects the samples to 5 N of force and 135 kPa tensile stress (~10^−6^ strain) during irradiation which is considered to have a negligible effect on the resulting radiation damage. The temperature gradient achieved between the two thermocouples on the sample was from 129.7 to 415.2 °C over the 15 mm separation, which corresponds to 9.7 W of heat being transferred along the sample. It has been assumed that thermal transfer is restricted to only conduction at these temperatures in vacuum and that the thermal gradient is linear along the sample length. Radiative losses and the small variation in thermal conductivity is neglected in the present study, however these effects require consideration for higher temperature experiments and different materials.

The above values suggest an approximate thermal conductance of 0.09 W/K and 0.08 W/K between the water coolant (estimated to be 16 °C) and the sample’s cold end, and between the heater at 550 °C and the sample’s hot end respectively. The clamped lengths at the cold and hot end were ~12 and ~7 mm respectively, which may have resulted in a slight reduction of the conductance at the hot end, however the thermal path differs substantially in the hot and cold clamps and without knowledge of the clamp temperatures, it is not possible to calculate the thermal transfer coefficient of the contacts.

During this work, multiple trial assemblies and irradiation experiments were conducted on identical samples and the temperatures measured at the hot and cold ends of the sample were both within 5 °C for the same heater temperature of 550 °C. Consistent measurements of hardness versus irradiation temperature were observed for all 4 samples irradiated, suggesting that the measured temperatures and temperature gradient in sample one was accurately reproduced in the other three samples. It is likely that this consistency was assisted by the relatively long clamping lengths for each sample. The temperature difference at the interface for each sample is likely affected by local surface roughness and variation in clamping force, however the influence of these factors becomes smaller as the clamping length is increased and the temperature of each sample approaches the clamp temperature.

Turning to the material property measurement, the CuCrZr exhibited a significant variation in irradiation hardening as a function of irradiation temperature, which transitioned from hardening to softening as the irradiation temperature increased, with a transition temperature of approximately 294 °C. It is generally accepted that hardening below 200 °C is due to the accumulation of irradiation induced defects (visible in the left of Fig. 4), which saturate at approximately 0.15 dpa at ~80–100 °C^22,23^, and that radiation enhanced recovery, recrystallisation or over-aging leads to irradiation softening above 300 °C^15^.

The strategy and validity of simulating neutron damage by charged particle irradiation is commonly debated^24–26^. However, the measurement of relative hardening in the present study demonstrated the same trend as that shown for neutron irradiated CuCrZr alloys as given by Fenici *et al*.^15^ despite the differences in alloy and multiple neutron irradiation conditions represented in their data. This suggests that nano-hardness after ion irradiation with the conditions used here is representative of neutron irradiation in this alloy.

Very little data is available on the microstructure of irradiated and as-received CuCrZr alloys. However, the APT measurement of particle size and number density is comparable to previous studies^27,28^. The composition measured by APT is also as expected for the aging condition^28^. For irradiated microstructures, at low temperature, (50 °C up to 0.2 dpa) no observable change in number density has been observed^29^, however the particle number density has been shown to vary with irradiation temperature. Singh *et al*.^30^ started with a similar peak-aged CuCrZr alloy (30 minutes at 475 °C = 2.9 nm particles, 0.59 × 10^23^ m^−3^) before irradiating to 0.3 dpa with neutrons (~70 days) at 100, 250 and 350 °C. Using TEM, they observed coarsening of the particles at all irradiation temperatures with a decrease in density below 300 °C and increase in particle number density at 350 °C to 1.8 × 10^23^ m^−3^. No data is currently available for alloys irradiated at higher temperatures. Therefore, there is no conclusive evidence in the literature for the irradiation softening to be caused by a change in particle distribution during the ion-irradiation.

As presented in Figs 5 and 7, no difference in particle size was observed in the cold un-irradiated C0 (as-received) and hot irradiated H1 conditions, however the material exhibited softening with a relative 20% reduction in hardness. This suggests that the irradiation softening is not due to a change in particle size or number density following irradiation. The H0 condition did show change in precipitate number density and size, exhibiting the conventional thermal ageing process of Oswald ripening. Therefore, our observations are comparable to those reported elsewhere but raises questions regarding the origin of irradiation softening. However, this is not the focus of this paper and a more extensive study will be discussed as part of future work elsewhere.

## Conclusions

A new technique in which samples subject to a thermal gradient are irradiated with charged particles has been developed. This technique delivers a relatively fast and inexpensive means of investigating the effect of temperature on the radiation response of materials, which is particularly useful for multiple applications from the validation of physics-based modelling and theory to the initial assessment of new/novel materials prior to further R&D investment.

The thermal gradient technique demonstrated here can be used on a wide range of materials of interest in the nuclear sector. The current work successfully demonstrated the technique on a copper alloy, which represents a more challenging application due to its high thermal conductivity and corresponding difficulty in producing a thermal gradient. Four identical samples were irradiated using the developed instrumentation and subsequent testing indicated that there was excellent consistency in nanoindentation hardness as a function of irradiation temperature. Facilitated by FIB lift-out specimen fabrication, TEM and APT were also used at selected irradiation temperatures highlighting the ability for further investigation at critical conditions identified on the hardness versus irradiation temperature relationship. The irradiation hardening measurements produced in this example agreed well with that available in the literature for a variety of similar alloys subjected to various neutron irradiation conditions. This result is significant; the cost and time taken to produce data using the thermal gradient technique is minute compared with that required to produce data using neutron irradiation. The thermal gradient technique offers a fast and relatively inexpensive technique to collect large amounts of data for the development of theory, and for engineering materials selection, prior to further investment using neutron irradiation. Furthermore, consistency in material and irradiation dosimetry is guaranteed, removing potential sources of experimental error.

The combination of microscale testing combined with ion irradiation was shown to yield irradiation property information as a function of irradiation temperature very rapidly. In addition to the techniques used here, several other post-irradiation techniques applicable to ion irradiated surfaces can be used to yield a wealth of data. These include the investigation of swelling by surface profilometry^31^, thermal conductivity by laser-induced transient grating techniques^32^, strain by X-ray diffraction^33^ and Raman/optical spectroscopy^34^ and primary defect populations by positron annihilation spectroscopy^35^. Such information is key to accelerating the timely development and optimisation of radiation-resistant materials for future fission and fusion power applications.

## Methods

### Material

A slab of CuCrZr was produced by Zollern GmbH & Co Material no. 2.1293 (in accordance with standards EN CW106C and UNS C18150). The material had a heat composition of 1.0 wt.% Cr, 0.06 wt.% Zr, <0.005 wt.% P, Cu bal., which was confirmed by X-ray Florescence Spectroscopy of the material in the water quenched state to be 0.83 wt.%Cr (±0.025 wt.%), Cu bal. where all other elements were below the limit of detection. Minor elements were detected using atom probe tomography: Zr 370 ± 190, N 240 ± 540, Li 80 ± 30 and Fe 130 ± 90 appm. These are the mean and one standard deviation of 9 measured specimens. The material was hot forged and solution heat treated at 970 °C ± 10 °C for approximately 20 minutes followed by water quenching. Following removal of the oxide scale, the final thickness of the slab was 35 mm, from which a block 90 × 130 × 35 mm was machined and aged at 480 °C (±10 °C) for 2 hours to produce a fine Cr-particle precipitation.

A Leco LM-100 microindentation hardness tester was used to measure the Vickers hardness of the CuCrZr material after heat treatment. A load of 100 gf was applied with a dwell time of 15 s. Sixteen indents were made with the indent impression areas all measured automatically post-test using the built-in optical microscope. These measurements were then checked and manually re-measured where necessary (e.g. if surface features influenced automatic edge detection). The average Vickers hardness result was 133.4 Hv (one standard deviation 5.06 Hv).

Four identical samples with dimensions of 35 × 1.2 × 1 mm^3^ were manufactured by electro-discharge machining, 0.6 mm diameter holes for thermocouples were drilled at 12.5 and 7.5 mm from the smallest faces and the samples were subsequently prepared by grinding and polishing on a 35 × 1 mm^2^ surface. Final polishing included a chemo-mechanical polish using a 0.04 µm colloidal silica suspension for approximately 10 minutes, followed by 5 minutes with hydrogen peroxide to neutralise the pH of the solution and prevent chemical bonding of silica particles to Cu.

### Irradiation

The irradiation was conducted at the laboratory for ion beam interactions in Ruđer Bošković Institute (RBI), Croatia. All 4 samples were clamped in a bespoke developed clamping device which is schematically drawn in Fig. 1a,c. The device consisted of two copper clamps which fixed on each end of four samples with two grub screws for each end of the sample. The ‘hot end’ clamp had a Pyrolytic Boron Nitride heater (Tectra, Germany) with embedded Pyrolytic Graphite element bolted to the assembly and the ‘cold end’ clamp was brazed to a 316 stainless steel pipe with an internal water-cooling channel. Insulated 0.5 mm diameter K-type thermocouples were inserted into the heater for control, and the hot and cold ends of one of the four samples for measurement. A thermal imaging camera (Optris Pi 640, Germany) was used to confirm the linearity of the thermal gradient along the length of the samples. The finished assembly is shown in Fig. 1b,d; this was inserted into the ‘Dual Beam Station for Fusion Materials – DiFU’ chamber, a new facility attached to the 6 MV tandem Van de Graaff and 1 MV Tandetron accelerators. The top of the assembly consists of a Conflat® CF63 rotatable flange which fixed to the top of the DiFU chamber, and the pipe length was designed so that the centre of the samples aligned with the centre of the ion beam.

The samples were subjected to a temperature gradient of 125 °C to 440 °C across the exposed area and irradiated with 2 MeV Cu^2+^ ions with a total beam current of 100 nA and current density of 30.4 nA/cm^2^ for 18.5 minutes. This corresponded to a total dose of 1.05 × 10^14^ ions/cm^2^ and 0.2 dpa at a dose rate of 1.8 × 10^−4^ dpa/s in pure Cu (peak damage), as calculated by SRIM^36^ using the Kinchin-Pease model and NRT calculation as suggested by Stoller *et al*.^37^ with a displacement energy of 30 eV for Cu^38^. A damage-depth plot is shown in the Supplementary Material, Fig. S1. The beam was scanned over a rectangular aperture and the 100 nA current was measured to be constant directly before and after the period of exposure by full collection in a 35 mm diameter, 70 mm deep faraday cup with electron suppression. The beam was scanned over the aperture at a rate of 488 Hz. Following irradiation, a Kapton polyimide film was placed over the samples and exposed to the beam to locate, assess uniformity and measure the exposed area; this was measured as 21.0 ± 0.2 mm in the vertical and 16.4 ± 0.2 mm in the horizontal dimension.

All four samples were marked by scoring a line with a scalpel on the irradiated surface, along the edges of the hot and cold clamps to provide a reference position from the location of the thermocouples on the measured sample with the clamping positions of each sample. The temperature at the scored lines of each sample was calculated for the measured sample by using the thermocouple data and assuming a linear temperature profile along the unclamped length of the sample.

### Nanoindentation and scanning electron microscopy

Nanoindentation was conducted with a G200 nanoindenter supplied by Keysight (formerly Agilent), with a Berkovich tip. The continuous stiffness measurement (CSM) technique with an amplitude and frequency of 2 nm and 45 Hz respectively was used and the tip geometry was calibrated before testing using fused silica reference sample in accordance with ref.^39^. Indents with a total depth of 1000 nm were produced along the length of each sample with a strain rate of 0.05/s, which provided a measurement of hardness in the damaged layer and un-irradiated substrate as the depth increases.

A Tescan Mira3 XMH scanning electron microscope (SEM) was used to image the samples. Samples were imaged post indentation to confirm the indent locations with respect to the scored lines on each sample. TEM and APT samples were prepared adjacent to selected indents with chosen values of hardness, using standard lift-out techniques^40^ with a FEI Helios 600 Dual-Beam Microscope.

Lift-outs were taken from one of the samples parallel to the length at positions corresponding to irradiation temperatures of 25, 166, 418 and 446 °C, designated as C0, C1, H1 and H0 respectively. The first and last positions where masked from the irradiation, the middle temperature positions were not.

### Microstructural characterisation: STEM

A FEI Talos F200X S/TEM with Super X Energy Dispersive X-rays detectors operated at 200 kV has been used to provide independent composition analyses of the Cr-rich precipitates in the CuCrZr TEM specimens. Energy Dispersive spectroscopy (EDS) spectrum images presented were generated by using the Bruker ESPRIT software and have the background subtracted using the standard build-in function available in the software.

### Microstructural characterisation: APT

Atom probe analyses were performed at 50 K using a LEAP-3000X HR instrument. A 532 nm wavelength laser with a 200 kHz pulse frequency, 0.5 nJ pulse energy and spot size of less than 10 µm was used to promote field evaporation. The standing voltage was maintained to give one detection event in every 100 pulses. Cr particles were identified in the data using the method of maximum separation distance of Cr ions only. Ions within a maximum separation (D_max_) of each other are considered clustered^41^. A D_max_ of 1.4 nm resulted in good classification of the particles, with the particle number not changing over a range of 0.9–1.8 nm. Only particles with a sufficient number of ions (N_min_) are counted, N_min_ was set to 50 to avoid the introduction of particles resulting from statistical fluctuations in the bulk. To extract the composition of the particles, an envelope distance of 0.7 and erosion distance of 0.5 nm were applied. The same particle selection parameters were used for all experimental volumes. Particle compositions are reported as the aggregate composition of all the particles extracted from one volume added together. Particle size is reported as $\sqrt[3]{3/4\pi \Omega \varepsilon }=0.265$ times the cube root number of Cr atoms, where $\Omega$ is the atomic volume of Cr (83.3 atoms/nm3) and $\varepsilon$ is the detection efficiency of the atom probe, assumed to be 0.37. The “matrix” composition was separated from the Cr particles using a selection with an N_min_ of 10 to detect any small particles or part-particles on the edge of the volume. For all composition measurements, background local subtraction were applied, but no mass-peak overlap solving was required.

## Supplementary information

Supplementary data processing Knitr script and data
