# Supplementary material for: Exploitation of thermal gradients for investigation of irradiation temperature effects with charged particles
Source: Sci Rep. 2019 Sep 19;9:13541. doi: 10.1038/s41598-019-49585-0 (PMC6753122; doi:10.1038/s41598-019-49585-0)
Supplement: Supplementary file 1 — Supplementary data processing Knitr script and data [file 41598_2019_49585_MOESM1_ESM.zip › SuplMater - Copy/SuplMater.pdf]

Supplementary Information: Exploitation of thermal gradients for investigation of irradiation temperature effects with charged particles

Chris Hardie*, Andrew J. London*, Joven J. H. Lim*,
Rob Bamber*, Tonci Tadic**, Marin Vuksic**, Stjepko Fazinic**

September 2, 2019

1 Introduction

Data analysis script for processing the nanoindentation (NI) data and atom probe particle sizes of ion-irradiated CuCrZr. This is a knitr (Rnw) document which takes source data (see data/ folder), runs analysis in the R programming language and generates a pdf output. The figures and table below are as shown in the main manuscript.

```
# required libraries avaliable from CRAN
library(data.table)
library(stringr)
library(dplyr)
library(xtable)
library(ggplot2)
library(readr)
library(ggpubr)
library(grid)
library(gridExtra)
library(purrr)
library(tidyr)
library(kableExtra)
library(broom)
```

```
# (Setup code chunk)
# set default figure options
knitr::opts_chunk$set(fig.align = "center",
                      fig.width = 5, fig.height = 3.5,
                      warning=FALSE, options(digits = 2))
options(digits = 2)

# font size for geom_text labels to be pt 8 size
font.size <- 8*0.3527777

## Custom functions
grid_arrange_shared_legend <- function(...) {
  plots <- list(...)
  g <- ggplotGrob(plots[[1]] + theme(legend.position="bottom"))$grobs
  legend <- g[[which(sapply(g, function(x) x$name) == "guide-box")]]
  lheight <- sum(legend$height)
  grid.arrange(arrangeGrob(grobs=lapply(plots, function(x)
      x + theme(legend.position="none")),ncol = 2),
    legend,
    ncol = 1,
    heights = unit.c(unit(1, "npc") - lheight, lheight))
}
```

1.1 SRIM damage prediction

Total ion flux per cm^2 is 1.05×10^{14}. Cu density in a volume (1 Angstrom x 1 cm^2) = 8.453×10^{14}. Depth units in the data are Angstrom.

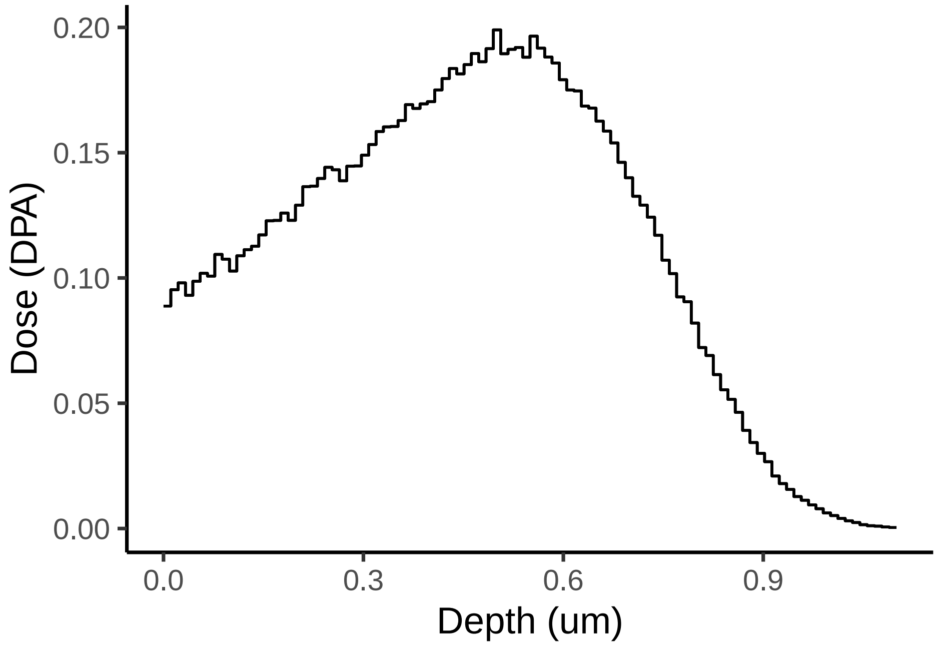

Figure S.1: SRIM predicted damage as a function of depth. See data/TRIM.IN for details.

```
VACANCY <- read_table2("data/SRIM_VACANCY.txt",
                       col_names = FALSE,
                       skip = 27,
                       col_types = cols(col_double(),
                                        col_double(),
                                        col_double(),
                                        col_character()),
                       n_max = 100)

ggplot(VACANCY, aes(X1/10000,(X2+X3)*1.05E14/8.453E14)) +
  geom_step(direction = "vh") +
  geom_step(aes((X1 - min(X1))/10000)) +
  theme_classic(14) +
  labs(x = "Depth (um)", y = "Dose (DPA)")
```

2 Experimental

Experiment ”RBI1”, four samples: _01–_04. Sample 01 had two thermocouples (TC) attached at ”Cold” and ”Hot” positions.

3 Data Analysis

Position information was obtained by SEM and optical measurements.

```
T.grad <- 306.6348591
T.inter <- 120.8869416

RBI1_stagePos <- read.csv("data/RBI1_01_stagePositions.csv")
RBI1_stagePos$T <- RBI1_stagePos$lcoord*T.grad + T.inter

LiftoutLocations <- mutate(subset(RBI1_stagePos, name %in% c("RBI1_01_Cold_Irrd_LiftOut",
                                                     "RBI1_01_Cold_Unirrd_LiftOut",
                                                     "RBI1_01_Hot_Irrd_LiftOut",
                                                     "RBI1_01_Hot_Unirrd_LiftOut")),
                           Y=c(2.76, 2.4, 2.13, 2.4),
                           label=c("C1","C0","H1","H0"),
```

```
                          dpa=paste("dpa:",c("0.39","0","0.39","0")),
                          Sample = "RBI1_01",
                          T = ifelse(T<25,25,T))
```

```
# Load data with temperature calculated from position
NI_RBI1 <- read_csv("data/NI_RBI_data.csv",
                    col_types = cols(
                      X1 = col_integer(),
                      Test = col_integer(),
                      AvgModulus = col_double(),
                      AvgHardness = col_double(),
                      DriftCorrection = col_double(),
                      Time = col_datetime(format = ""),
                      TipName = col_character(),
                      Temperature = col_double(),
                      Xposition = col_double(),
                      Yposition = col_double(),
                      Usable = col_integer(),
                      Sample = col_character(),
                      lcoord = col_double(),
                      dpa = col_character(),
                      HotX = col_double(),
                      ColdX = col_double(),
                      T = col_double()
                    ))
```

```
NonIrrdHAvg <- subset(NI_RBI1,Usable==1 &
         (T<350 & dpa=="0")) %>%
  summarise(H=mean(AvgHardness),
            H.sd=sd(AvgHardness),
            N=length(AvgHardness))
```

Average and standard deviation of the non-irradiated (but heat treated) side: 2.42 ± 0.22, N = 154

3.1 Figure 2

Plot of hardness and Young's modulus for heat-treated (not irradiated) and irradiated specimens.
First the Hardness plot is made...

```
H_plot <- ggplot(mutate(subset(NI_RBI1,Usable==1 & !(T<160 & dpa=="0.39")),
                        Y=AvgHardness,
                        dpa = paste("dpa:",dpa)),
       aes(T,
           Y,
           colour=substr(Sample,7,8),
           shape=substr(Sample,7,8)))+
  annotate("segment", x=80, xend=500, y=2.42,yend=2.42, colour="grey95", size=2) +
  geom_point(size=1)+
  labs(y="Hardness (GPa)",
       x = expression(Temperature~(degree*C)),
       colour="Sample",
       shape="Sample") +
  theme_pubr(8) +
  theme(strip.background = element_rect(colour=NA, fill=NA),
        legend.position = "bottom",
        panel.spacing = unit(1, "lines"))+
  scale_color_brewer(palette = "Spectral")+
  facet_grid(dpa~.) +
  geom_text(data = LiftoutLocations,
            aes(label=label),
            show.legend = FALSE,
```

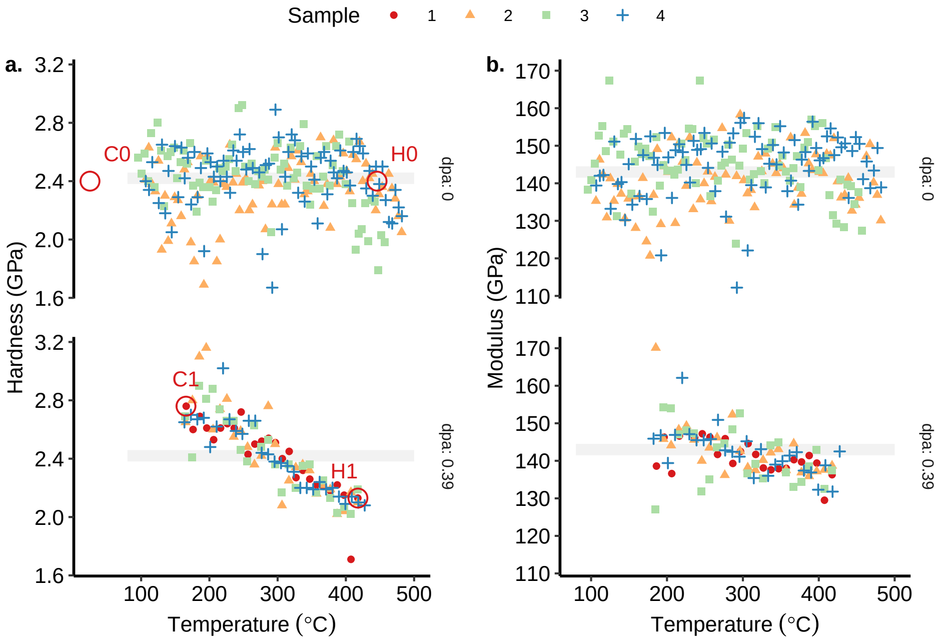

Figure 2: Comparison of irradiated and heat-treated only nano-indentation results. Each colour/shape shows a different irradiated sample. Grey band shows as-received result.

```
            vjust = -1.3,
            hjust=c(0.5,-0.5,1,-0.5),
            size = font.size) +
  geom_point(data = LiftoutLocations,
             shape = 1,
             size = 3,
             show.legend = FALSE)
```

... then the plot of modulus.

```
M_plot <- ggplot(mutate(subset(NI_RBI1,Usable==1 & !(T<180 & dpa=="0.39")),
                        Y=AvgModulus,
                        dpa = paste("dpa:",dpa)),
       aes(T,
           Y,
           colour=substr(Sample,7,8),
           shape=substr(Sample,7,8)))+
  annotate("segment", x=80, xend=500, y=143,yend=143, colour="grey95", size=2) +
  geom_point(size=1)+
  labs(y="Modulus (GPa)",
       x = expression(Temperature~(degree*C)),
       colour="Sample",
       shape="Sample") +
  theme_pubr(8) +
  theme(strip.background = element_rect(colour=NA, fill=NA),
        legend.position = "bottom",
        panel.spacing = unit(1, "lines"))+
  scale_color_brewer(palette = "Spectral")+
  facet_grid(dpa~.)
```

The two plots (two panels each) are arranged using ggarrange.

```
ggarrange(H_plot,M_plot, ncol=2, common.legend = TRUE,
          labels=c("a.","b."), font.label = list(size=8))
```

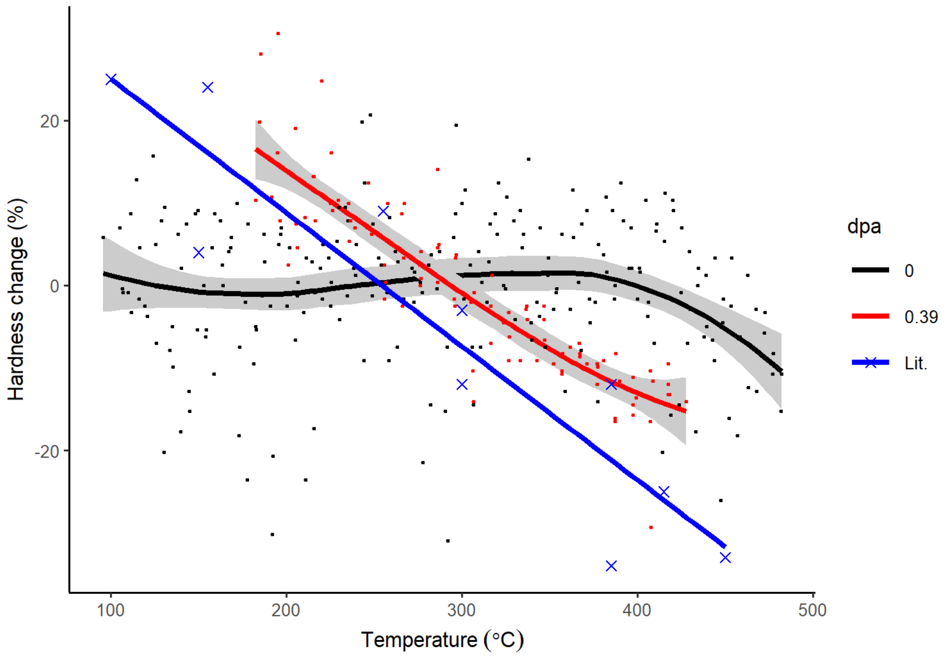

Figure 3: Comparison of hardness data with yield strength data from [10.1016/0022-3115(94)90093-0].

```
#grid_arrange_shared_legend(H_plot,M_plot)
```

3.2 Figure 3

Loess lines of best fit and literature data comparison. The NI data above is plotted and fitted all at once.

```
NIfit <- ggplot(subset(NI_RBI1,Usable==1 & !(T<180 & dpa=="0.39")),
       aes(T,100*(AvgHardness-2.42)/2.42,colour=dpa)) +
  geom_smooth(show.legend = FALSE, fill="grey80", alpha=1) +
  geom_point(size=0.2)+
  labs(y="Hardness change (%)", x = expression(Temperature~(degree*C))) +
  scale_colour_manual(values = c("black","red","blue")) +
  theme_classic(8)
```

Compare with literature values from [10.1016/0022-3115(94)90093-0].

```
hardnessLit <- structure(list(
  Tirrd = c(450L, 385L, 385L, 150L, 300L, 300L, 100L, 415L, 155L, 255L),
  dH = c(-33L, -12L, -34L, 4L, -3L, -12L, 25L, -25L, 24L, 9L)),
  .Names = c("Tirrd", "dH"),
  class = "data.frame",
  row.names = c(NA, -10L))

NIfit +
  geom_point(data=hardnessLit,aes(Tirrd,dH,colour="Lit."), shape = 4)+
  geom_smooth(data=hardnessLit,aes(Tirrd,dH,colour="Lit."),method="lm",se=FALSE) +
  guides(colour = guide_legend(override.aes = list(shape = c(NA,NA,4))))

## `geom_smooth()` using method = 'loess' and formula 'y ~ x'
```

3.3 Table 1

Find the linear fit of the data:

```r
# SElect only the irradiated data, and work out the dH
NI_data <- mutate(subset(NI_RBI1,Usable==1 & !(T<160 & dpa=="0.39") & dpa!=0),
                  dH = AvgHardness-2.42)

lmFit_data <- NI_data %>%
  nest(-Sample) %>%
  mutate(
    fit = map(data, ~ lm(dH~T, data = .x)),
    tidied = map(fit, tidy)
  ) %>%
  unnest(tidied) %>% select(Sample, term, estimate, std.error)

lmFit_tab <- data.table::dcast(setDT(lmFit_data),
                               formula=Sample~term, value.var=c("estimate","std.error"))
colnames(lmFit_tab) <- c("Sample","C","M","Cerr","Merr")

# Fit all the data as well
fitAllNI <- lm(data=NI_data, dH~T)
f <- summary(fitAllNI)
fitAllNI.dt <- data.frame(Sample="All",
                          C=f$coefficients[1,1],
                          M=f$coefficients[2,1],
                          Cerr=f$coefficients[1,2],
                          Merr=f$coefficients[2,2])

lmFit_table <- bind_rows(lmFit_tab, fitAllNI.dt) %>%
  mutate(T0 = -C/M, T0_err=(-C/M)*sqrt((Cerr/C)^2+(Merr/M)^2))
```

Table 1 in the manuscript: linear fit data for each specimen and for all specimens.

```r
mySan <- function(x){
  x1 <- x
  x1[!str_detect(x,"\\$")] <- str_replace_all(x[!str_detect(x,"\\$")], fixed("_"), "\\_")
    return(x1)
  }
print(
  xtable(lmFit_table %>%
         mutate(T0 = -C/M, T0_err=(-C/M)*sqrt((Cerr/C)^2+(Merr/M)^2)) %>%
         transmute(
           Sample=Sample,
           Intercept=sprintf("%2.2f $\\pm$ %2.2f",C,Cerr),
           Gradient=sprintf("%2.4f $\\pm$ %2.4f",M,Merr),
           `$T_x$`=sprintf("%2.0f $\\pm$ %2.0f",T0,T0_err)
           )
       ),
  booktabs = TRUE, sanitize.text.function=mySan)
```

	Sample	Intercept	Gradient	T_x
1	RBI1_01	0.80 ± 0.08	-0.0028 ± 0.0003	290 ± 43
2	RBI1_02	1.05 ± 0.13	-0.0035 ± 0.0004	301 ± 51
3	RBI1_03	0.87 ± 0.10	-0.0030 ± 0.0003	291 ± 47
4	RBI1_04	0.81 ± 0.08	-0.0028 ± 0.0003	294 ± 39
5	All	0.88 ± 0.05	-0.0030 ± 0.0002	294 ± 23

3.4 Cluster data

All the information resulting from the particle-identification is stored in the CuCrZr_clusterData.csv file, this is read and the number of Cu atoms used to define the particle size. Run R14_26450 is excluded.

```
CuCrZr_clusterData <- read_csv("data/CuCrZr_clusterData.csv")

## Parsed with column specification:
## cols(
##   .default = col_double(),
##   ROI = col_character(),
##   `Ion(s)` = col_character(),
##   ID = col_character(),
##   mat = col_character()
## )
## See spec(...) for full column specifications.

# reorder mat factors
CuCrZr_clusterData$mat <- factor(CuCrZr_clusterData$mat,
                                 levels=c("cold ref", "cold irrd", "hot irrd", "hot ref"),
                                 labels=c("C0", "C1", "H1", "H0"))
```

3.5 Figure 7

Code chunk for generating Figure 7 of the manuscript.

```
# averaged data for vlines
mu <- subset(CuCrZr_clusterData,ID!="R14_26450") %>%
  group_by(mat) %>%
  summarise(grp.med = median(0.26*Cr^(1/3)),
            grp.mean = mean(0.26*Cr^(1/3)))

ggplot(subset(CuCrZr_clusterData,ID!="R14_26450"),aes(x=0.26*Cr^(1/3),fill=mat)) +
  geom_histogram(binwidth = 0.1) +
  theme_pubr(8)+theme(strip.background = element_blank(), legend.position = "none") +
  labs(y="Count",x="Particle Radius (nm)") +
  scale_fill_brewer(type="seq",palette = "Spectral", direction = -1) +
  scale_shape_manual(values=1:9) +
  facet_grid(mat~.) +
  geom_vline(data=mu,aes(xintercept=grp.mean)) +
  scale_y_continuous(breaks = c(0,10,20))
```

4 Appendix

Display the session info for reproducibility.

```
sessionInfo()

## R version 3.5.1 (2018-07-02)
## Platform: x86_64-w64-mingw32/x64 (64-bit)
## Running under: Windows 10 x64 (build 17134)
##
## Matrix products: default
##
## locale:
## [1] LC_COLLATE=English_United Kingdom.1252
## [2] LC_CTYPE=English_United Kingdom.1252
## [3] LC_MONETARY=English_United Kingdom.1252
## [4] LC_NUMERIC=C
## [5] LC_TIME=English_United Kingdom.1252
##
## attached base packages:
```

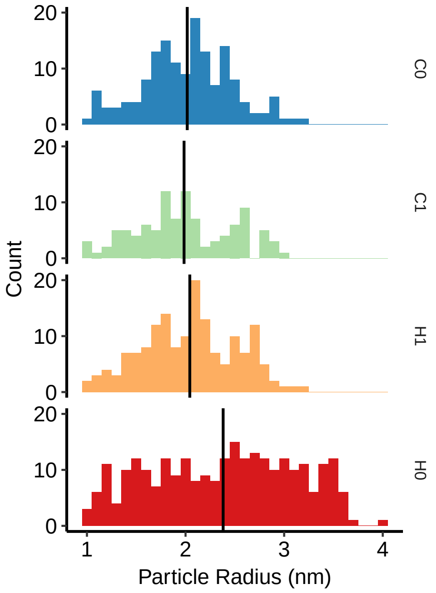

Figure 7: Cr Cluster size histograms for each sample condition. Vertical lines mark the median of each distribution

```
## [1] grid      stats     graphics  grDevices utils     datasets  methods
## [8] base
##
## other attached packages:
##  [1] bindrcpp_0.2.2    broom_0.5.1       kableExtra_0.9.0
##  [4] tidyr_0.8.2       purrr_0.2.5       gridExtra_2.3
##  [7] ggpubr_0.2        magrittr_1.5      readr_1.3.0
## [10] ggplot2_3.1.0     xtable_1.8-3      dplyr_0.7.8
## [13] stringr_1.3.1     data.table_1.11.8 knitr_1.21
##
## loaded via a namespace (and not attached):
##  [1] Rcpp_1.0.0         RColorBrewer_1.1-2 highr_0.7
##  [4] pillar_1.3.0       compiler_3.5.1     plyr_1.8.4
##  [7] bindr_0.1.1        tools_3.5.1        digest_0.6.18
## [10] lattice_0.20-35    nlme_3.1-137       viridisLite_0.3.0
## [13] evaluate_0.12      tibble_1.4.2       gtable_0.2.0
## [16] pkgconfig_2.0.2    rlang_0.3.0.1      rstudioapi_0.8
## [19] xfun_0.4           xml2_1.2.0         httr_1.4.0
## [22] withr_2.1.2        generics_0.0.2     hms_0.4.2
## [25] cowplot_0.9.3      tidyselect_0.2.5   glue_1.3.0
## [28] R6_2.3.0           rmarkdown_1.11     reshape2_1.4.3
## [31] backports_1.1.2    scales_1.0.0       htmltools_0.3.6
## [34] rvest_0.3.2        assertthat_0.2.0   colorspace_1.3-2
## [37] labeling_0.3       stringi_1.2.4      lazyeval_0.2.1
## [40] munsell_0.5.0      crayon_1.3.4
```
